# High-Performance Ag-NWs Doped Graphene/ITO Hybrid Transparent Conductive Electrode

**DOI:** 10.3390/mi16020204

**Published:** 2025-02-11

**Authors:** Hana Bourahla, Susana Fernández, Yu Kyoung Ryu, Andres Velasco, Chahinez Malkia, Alberto Boscá, M. Belén Gómez-Mancebo, Fernando Calle, Javier Martinez

**Affiliations:** 1 Instituto de Sistemas Optoelectrónicos y Microtecnología (ISOM-UPM), E.T.S.I. de Telecomunicación, Universidad Politécnica de Madrid, Av. Complutense 30, 28040 Madrid, Spainandres.velasco@upm.es (A.V.); fernando.calle@upm.es (F.C.); 2 Departamento de Ingeniería Electrónica, E.T.S.I. de Telecomunicación, Universidad Politécnica de Madrid, Av. Complutense 30, 28040 Madrid, Spain; 3 Departamento de Energía, Centro de Investigaciones Energéticas, Medioambientales y Tecnológicas (CIEMAT), Avda. Complutense 40, 28040 Madrid, Spain; susanamaria.fernandez@ciemat.es; 4 Departamento de Física Aplicada e Ingeniería de Materiales, E.T.S.I. Industriales, Universidad Politécnica de Madrid, C/José Gutiérrez Abascal 2, 28006 Madrid, Spain; 5 División de Química, Centro de Investigaciones Energéticas, Medioambientales y Tecnológicas (CIEMAT), Avda. Complutense 40, Madrid 28040, Spain; 6 Departamento de Ciencia de Materiales, E.T.S.I Caminos, Canales y Puertos, Universidad Politécnica de Madrid, C/Profesor Aranguren s/n, 28040 Madrid, Spain

**Keywords:** transparent conductive electrodes, graphene/ITO hybrid, silver nanowires, sheet resistance

## Abstract

Indium tin oxide (ITO) is a commonly used material for transparent conductive electrodes (TCE) in optoelectronic applications. On the other hand, graphene has superior electrical conductivity and exceptional mechanical flexibility, which makes it a promising candidate as a TCE material. This work proposes a CVD graphene/ITO hybrid electrode enhanced by doping with silver nanowires (Ag-NWs). The study aims to improve the performance of the electrode by optimizing two key parameters during the fabrication process: the thermal annealing time after the transfer of graphene on ITO and the Ag-NWs doping conditions. The annealing treatment is fundamental to reducing the residues on the surface of graphene and increasing the interface contact between graphene and ITO. The correct coverage and distribution of the dopant on graphene is obtained by controlling the concentration of the Ag-NWs and the spin coating speeds. The results indicate a substantial improvement in the optical and electrical performance of the Ag-NWs/graphene/ITO hybrid electrode. A remarkably low sheet resistance of 42.4 Ω/sq (±2 Ω/sq) has been achieved while maintaining a high optical transmittance of 87.3% (±0.5%).

## 1. Introduction

Transparent conductive electrodes (TCEs) are crucial components in various applications, including touch screens, LEDs, solar cells, and optoelectronic devices [1,2]. Both high optical transmittance and high electrical conductivity are the main required characteristics to achieve a high-performance TCE [3]. Several materials that present those characteristics are used for transparent conductive electrodes, such as transparent conductive oxides (TCOs). Indium tin oxide (ITO) is currently the most used commercial TCO material for this application due to its favorable optical and electrical properties [4,5,6]. On the other hand, graphene, a monolayer of carbon atoms with a honeycomb structure, has emerged as an ideal candidate for transparent conductive electrodes in optoelectronic devices, particularly in solar cell applications [2,7,8,9]. It has a high electrical conductivity of up to 1.5 × 10^6^ S/m [10], a high optical transmittance of 97.7%, and exceptional mechanical properties [11,12]. There are several methods for obtaining graphene, including mechanical exfoliation [13], chemical exfoliation [14], epitaxial growth [15], and chemical vapor deposition (CVD) [16] on a catalytic metal template. Among these techniques, CVD stands out as the most effective approach for the large-scale production of high-quality graphene [17,18]. However, the transfer methods of CVD-grown graphene to the target substrate create wrinkles and cracks in the graphene film, which decreases the electrical conductivity of the monolayer of graphene [19]. Another issue lies in the fact that the most commonly employed polymer in this process, polymethyl methacrylate (PMMA), is not completely removed after the transfer of graphene on the target substrate due to the strong covalent bonds formed between PMMA and graphene. This incomplete removal of PMMA can lead to damaging effects such as unintentional doping levels and decreased electrical conductivity [20,21,22,23].

Recently, various methods have been investigated to improve graphene’s properties, such as hybridizing graphene with other materials, optimizing the graphene transfer process, and chemical doping of the graphene [7]. In fact, a few studies have reported the hybridization of graphene with ITO material. It is found that depositing a graphene layer on the ITO surface has improved the electrical properties of both materials [18,24]. For the chemical doping, carbon nanotubes (CNTs) [25,26,27], conductive polymers [28,29,30], metallic nanoparticles (NPs), and metal nanowires (NWs) [31,32,33,34] have been used as dopants to enhance the properties of graphene [28,35]. Among them, silver nanowires (Ag-NWs) have gained significant attention for their potential applications as transparent conductive electrodes, owing to their optical transparency, high electrical conductivity, and high flexibility [32,36]. In addition to their simple synthesis methods, their solution processing and compatibility with various wet coating methods facilitate their integration with monolayer CVD graphene [37].

Since optoelectronic technology is still based on the ITO as the main transparent conductive electrode, despite some material limitations, several studies were performed to explore the performance of hybrid TCEs based on graphene/ITO [38]. This present work has the target to combine the suitable properties of ITO, graphene, and Ag-NWs to address their individual limitations while enhancing the optical and electrical performance of the hybrid TCE. To accomplish this objective, a simple and easy strategy was followed based on refining the TCE fabrication process by implementing thermal annealing (TA) treatment for different time intervals, which helps to enhance the material’s quality [39]. Additionally, we introduced silver nanowires (Ag-NWs) onto the top surface of the graphene layer to further boost its conductivity and transparency. This dual approach aimed to maximize the performance of the hybrid TCEs in various technological applications. The thermal annealing treatment was double-fold: it removed the resist residue on the top surface of the graphene and improved the interface contact between the graphene bottom surface and the ITO film. Several studies have reported that annealing graphene at a high temperature (around 400 °C), after transferring it onto the target substrate, can effectively remove the impurities and resist residues present on the graphene surface after growth and transfer [40,41,42,43]. And improve the structure, optical, and electrical properties of ITO film [44]. However, this high temperature is not suitable for silicon heterojunction solar cells, where this graphene/ITO hybrid TCE could be potentially used. The fabrication of the heterojunction solar cell including the deposition of the hybrid TCE, is commonly carried out using plasma-enhanced chemical vapor deposition, which requires a low processing temperature of no more than 200 °C [45,46,47]. Therefore, an optimization of the annealing process at low temperatures by adjusting the treatment time was necessary to improve the quality of the TCEs without undermining the enhancements introduced by other fabrication steps. Furthermore, we chose the incorporation of Ag-NWs on the graphene layer to improve the electrical conductivity of the hybrid TCE. We observed that the density of the dopants and the speed of the spin coating under which they were deposited on graphene had a significant impact on the electrical and, notably, the optical characteristics of the proposed TCE. Consequently, this work focused on optimizing these conditions to achieve the most favorable outcomes, which have been a low sheet resistance of 42.4 Ω/sq (±2 Ω/sq) while maintaining a high optical transmittance of 87.3% (± 0.5%).

## 2. Methods and Materials

### 2.1. CVD Graphene Synthesis

The monolayer graphene film is synthesized using a low-pressure chemical vapor deposition (LPCVD) technique with a cold-wall CVD reactor (Aixtron Black Magic Pro, Herzogenrath, Germany). Uncoated commercial copper foil (25 μm thick, 99.8% purity), purchased from Alfa Aesar (Darmstadt, Germany), is used as the catalytic metal film. A 6 × 5 cm Cu foil is electro-polished for 10 min in 55 wt.% phosphoric acid (H_3_PO_4_) solution. Then, a wet oxidation step in hot hydrogen peroxide (H_2_O_2_, 30% concentration) is performed at 80 °C for 10 min. The Cu foil is then loaded inside the CVD chamber, which is connected to all the process gases, including argon (Ar), hydrogen (H_2_), and methane (CH_4_). The temperature is raised to 1050 °C under an Ar atmosphere in the next step. The growth of graphene is then initiated by introducing a mixture of CH_4,_ H_2_, and Ar (0.08%, 0.74%, and 69.17% in volume, respectively) for 20 min at 50 mbar pressure.

### 2.2. Indium Tin Oxide Growth

The ITO thin films are fabricated in a commercial UNIVEX 450B system (Leybold, Cologne, Germany), equipped with four magnetron sources placed in a confocal geometry concerning the substrate holder, distanced from each other about 15 cm. This confocal configuration helps to ensure homogeneity in the deposited thin film. Two of the four magnetrons are operated by radio frequency (RF), and the other ones by direct current (DC). Specifically, a 4-inch diameter SnO_2_:In_2_O_3_ (90/10%wt) ceramic target (Neyco, Vanves, France) is placed on a direct current (DC) source. The sputtering process of the ITO thin films is carried out at a base pressure of 10^−5^ Pa in an oxygen-free environment and with no intentional substrate heating. The process gas used in the deposition of ITO is Ar with a purity of 9N5, whose flux set to 5 sccm is controlled with a mass flow controller (MKS, Andover, MA, USA), which corresponds to a working pressure of 0.17 Pa. The DC power is set to 25 W and the deposition time to 50 min to have a thickness of 80 nm. During the entire sputtering process, the substrate is rotated at a speed of 20 rpm.

### 2.3. Graphene/ITO-Based Transparent Conductive Electrode Fabrication

In this study, ITO film is grown on both silicon and glass substrates for the following reason: the hybrid graphene/ITO TCE is fabricated on the silicon substrate because the goal of the present work is the optimization of its properties to use it later in silicon heterojunction solar cells. The growth of the samples on the glass substrate is performed to enable the optical transmittance measurements of the hybrid graphene/ITO electrode.

An automatic transfer system [17] is used to transfer the monolayer graphene onto ITO/silicon and ITO/glass target substrates. The graphene/copper foil is cut into 18 mm diameter pieces after being coated with a 0.25 µm thick layer of PMMA using spin coating at 3000 rpm for 60 s. Then, the backside copper is etched using a 0.3 M ammonium peroxydisulfate (APS) etchant solution. After 3 h of etching with APS and 6 h of cleaning with DIW, the PMMA/MLG stack is automatically transferred onto the substrates and then dried in a vacuum overnight to remove the residual DIW. Then, the PMMA layer is removed by immersing the sample in hot acetone at 55 °C for 20 min, then rinsing with IPA and DIW and drying with nitrogen [17].

A thermal annealing treatment is applied on graphene/ITO deposited in both silicon and glass substrates at a low temperature of 150 °C varying the annealing times: 15 min, 60 min, and 120 min. The annealing process is conducted under a controlled atmosphere using an inert gas, Nidron (95.5% of N_2_ + 4.5% of H_2_), at 30–35 sscm in a conventional oven [43,45]. For the doping treatment, a solution of silver nanowires in IPA is purchased from Sigma Aldrich. The diameter, length, and density of silver nanowires are, respectively, 70 nm (±10 nm), 40 μm (±5 μm), and 5 mg/mL. Two different concentrations of Ag-NWs, 1 mg/mL and 2 mg/mL are prepared by diluting the initial density in IPA and sonicating for several minutes. The Ag-NWs are deposited on the samples by spin coating at two different speeds of 1000 rpm and 3000 rpm for 1 min and then baked straightaway at 100 °C for 2 min on a hot plate under ambient conditions to evaporate IPA residues and ensure good contact between the Ag-NWs and graphene [48,49,50].

**Figure 1:**
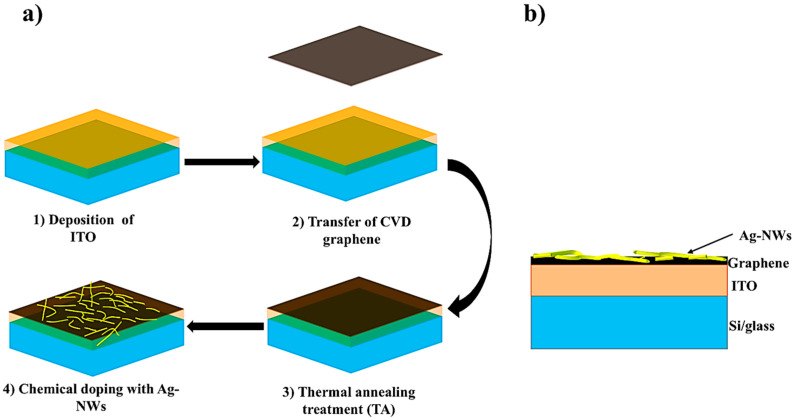
(**a**) Schematic diagram with the fabrication process steps of the transparent conductive electrode presented in this work. (**b**) Scheme of the cross-section of the sample at the end of the fabrication steps.

Figure 1a summarizes the different fabrication steps of the samples: (1) growth of the ITO film on Si or glass substrates, (2) transfer of a monolayer CVD graphene on the ITO layer, (3) thermal annealing treatments of the samples, and (4) doping of graphene with silver nanowires. Figure 1b represents the scheme of the cross-section of a finalized device. The samples are electrically and optically characterized after each step of the process to follow the evolution of the sheet resistance, optical transmittance, and reflectance of the samples.

The different samples studied in the present work are shown in Table 1, as a function of the target substrate, annealing times, and doping conditions.

### 2.4. Characterization Techniques

To identify the quality and the chemical composition of graphene material, Raman spectroscopy was conducted by using a commercial LabRAM HR Evolution microscope (Horiba, Kyoto, Japan), equipped with an excitation laser with a wavelength of 532 nm. The measurements were performed using a grating of 600 gr/mm and a confocal hole of 50 µm, with a 100× magnification objective, 20 s of integration time, and 5 accumulations. The spectra were taken in the range from 1000 to 3000 cm^−1^. To study the morphology of the Ag-NWs deposited on the devices, a scanning electronic microscope (SEM) FEI inspect F50 (FEI, Hillsboro, OR, USA) was used. The hybrid transparent conductive electrodes’ electrical performance was evaluated using a semi-automatic Four Point Probe Measurement System SIGNATURE S-303-8 to perform sheet resistance and bulk resistivity measurements (Signatone Corporation, Gilroy, CA, USA). The optical transmittance and reflectance were measured with a commercial Lambda 1050 spectrophotometer (Perkin Elmer, Waltham, MA, USA), at normal incidence and room temperature (RT) in the wavelength range of 200—2500 nm.

## 3. Results and Discussions

### 3.1. Thermal Annealing Time Effect on the Electrical and Optical Properties of Hybrid Graphene/ITO Electrode

**Figure 2:**
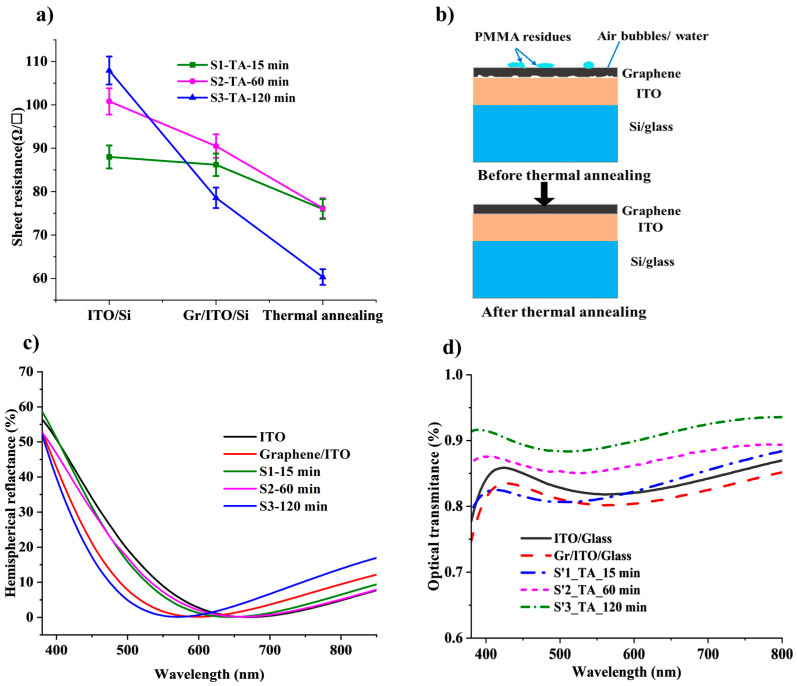
(**a**) Sheet resistance variation in the hybrid graphene/ITO electrode on/Si substrate at different process steps for the three thermal annealing times. (**b**) A scheme with cross-sections explains the effect of the thermal annealing step on the removal of the PMMA residues and air bubbles or water confined between the graphene layer and ITO. The elements of the scheme are not in scale. (**c**) Hemispherical reflectance of the hybrid graphene/ITO electrode deposited on Si substrate at different thermal annealing times. (**d**) Normalized optical transmittance of the hybrid graphene/ITO electrode deposited on the glass substrate at different process steps for the three different thermal annealing times. wavelength.

The electrical performance of the hybrid transparent conductive electrodes is evaluated by measuring the sheet resistance (R_s_) as a function of different annealing times. Figure 2a shows the sheet resistance values as a function of the TA times from samples S1, S2, and S3 for the silicon substrate (the names of the samples and their descriptions can be found in Table 1. The sheet resistance of the hybrid stack is improved after transferring the graphene monolayer onto ITO/silicon substrates. Initially, the values of the sheet resistance are around 91.7, 100.8, and 107.9 Ω/sq (±5 Ω/sq) for ITO/Si samples S1, S2, and S3, respectively. After the deposition of the graphene monolayer, the sheet resistance of the graphene/ITO hybrid decreased to 85.6, 90.5, and 73.7 Ω/sq (±2 Ω/sq), respectively. Table S1, found in the Supplementary Information file, summarizes the R_s_ values after the deposition of MLG on top of ITO/Si and after different thermal annealing times and various Ag-NWs doping conditions. These results can be explained as follows. The amount of electrical conductivity in a material is directly proportional to the mobility and concentration of charge carriers. It is reported that when comparing graphene to ITO, it is found that graphene has a higher carrier mobility but a lower carrier density (concentration) than ITO [51,52]. Therefore, by contacting graphene with ITO, the hybrid electrode presents both better carrier concentration and carrier mobility with respect to the individual electrode by introducing n doping from foreign material [18]. After applying the thermal annealing at 150 °C, for three different times, 15 min (S1), 60 min (S2), and 120 min (S3), the sheet resistance of the graphene/ITO/Si stack showed better improvement. It decreased to 73.3 Ω/sq, 78.2 Ω/sq, and 59.6 Ω/sq (±2 Ω/sq), respectively. Thus, the annealing treatment enhanced the electrical performance of the hybrid electrode for all the tested times, achieving the best result of a 19% decrease in the R_S_ value for the longest time of 120 min (S3). After transferring the graphene, it is difficult to completely remove PMMA from the graphene surface [53], resulting in the presence of residues that negatively affect the electrical performance of the graphene [43,44,54]. Therefore, the TA treatment after the transfer of graphene leads to the elimination and evaporation of the PMMA residues [42,55]. Furthermore, the interface between graphene and ITO improves as well, due to the elimination of trapped moisture and air and the enhancement in the contact area of both surfaces under the application of heat [56], as illustrated in the scheme from Figure 2b.

The optical performance of the hybrid graphene/ITO TCEs was evaluated by measuring their optical reflectance and normalized transmittance on both silicon (S1, S2, S3) and glass (S’1, S’2, S’3) substrates, as displayed in Figure 2c,d, respectively. First, analyzing Figure 2c, the optical reflectance of the sample after the deposition of graphene (red curve) showed a small decrease compared to the as-deposited ITO on a silicon substrate (black curve). The optical reflectance of the ITO/Si sample exhibited an initial value of 11.9%. However, after the deposition of a graphene film, this value decreased to 8.4%, demonstrating a substantial enhancement in anti-reflective (AR) properties. The presence of graphene on top of the ITO layer significantly improves its ability to minimize reflection compared to using the ITO layer alone. This advancement in optical performance is particularly encouraging for its potential application as a transparent conducting electrode in silicon heterojunction technology [57]. Following the annealing process, the reflectance measurements revealed interesting trends among the samples. Samples S1 and S2, subjected to thermal annealing for 15 and 60 min, respectively, exhibited reflectance values of 11.2% and 10.7%. These values closely resembled the as-deposited ITO/Si sample, indicating that the annealing had little effect on their reflective properties. In contrast, sample S3, which had undergone graphene transfer, revealed a similar behavior to that observed right after the transfer, with a reflectance measurement of 8.6%. This indicates that the presence of the graphene layer continues to provide enhanced anti-reflective qualities even after the annealing process [58,59].

The optical transmittance was measured using the samples deposited on glass (Figure 2d), as explained in the experimental section. It was normalized with the optical transmittance of the glass. The initial optical transmittance of ITO/glass was 83.5% in the visible region. After the transfer of the graphene monolayer, the optical transmittance decreased by about 1.5% to achieve 82.0% for MLG/ITO/glass, which indicates the deposition of the graphene monolayer [60]. After applying the TA treatment to the samples at various times, the transmittance increased again. By increasing the TA time, the optical transmittance increased, as Figure 2d depicts. An optical transmittance of 90.7% was achieved with a thermal annealing time of 120 min (S’3) compared to 83.4% and 87.7% for the annealing times of 15 (S’1) and 60 (S’2) min, respectively (see Table S2 in the Supplementary Information file). The findings reveal that subjecting the graphene/ITO hybrid to an annealing treatment at 150 °C for a prolonged period of 120 min significantly enhances its optical performance. This enhancement is achieved by effectively removing impurities that accumulate on the surface of the graphene during the chemical vapor deposition (CVD) growth process. Additionally, the treatment evaporates the residual PMMA left after the transfer process, resulting in a cleaner and more efficient material. Such improvements in surface quality contribute to optimized optical characteristics, making the graphene/ITO hybrid electrode more suitable for various applications [53,61].

### 3.2. Silver Nanowires Doping Characterization

To prove the effect of silver nanowire doping on graphene, a preliminary study was conducted on graphene field-effect transistor (GFET) devices. Metallic electrodes were deposited on a silicon oxide/Si substrate, and a piece of graphene S311 was transferred on them, obtaining a back gated FET device. The GFET was annealed at 400 °C for 10 min (the thermal annealing temperature and time were optimized for our GFET in a previous work). Doping with silver nanowires was performed with Ag-NWs with a concentration of 1 mg/mL and by spin coating at 1000 rpm for 1 min. The GFET was characterized by a probe station and semiconductor analyzer, and the data were treated with a Collab program to evaluate the sheet resistance of graphene. Figure S1 (supplementary information file) shows the histogram of the sheet resistance of the GFET as fabricated, after annealing, and after doping with Ag-NWs. The as-fabricated GFET showed a sheet resistance of 1400 Ω/sq, which decreased to 1150 Ω/sq after thermal annealing. Doping with Ag-NWs significantly improved the sheet resistance of graphene, resulting in a 70% decrease. The sheet resistance was reduced from 1150 Ω/sq after thermal annealing to 335 Ω/sq after doping with Ag-NWs. This result confirms the significant effect of Ag-NWs on enhancing the electrical performance of the graphene.

**Figure 3:**
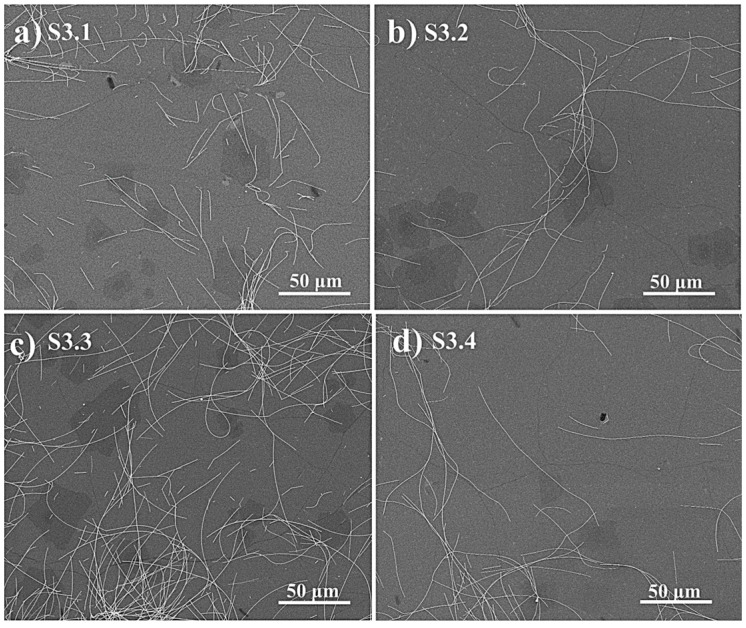
SEM images of graphene/ITO electrodes (sample S3) doped with silver nanowires at different conditions: (**a**) Sample S3.1, 1 mg/mL of density, spin-coated at 1000 rpm. (**b**) Sample S3.2, 1 mg/mL of density, spin-coated at 3000 rpm. (**c**) Sample S3.3, 2 mg/mL of density, spin-coated at 1000 rpm. (**d**) Sample S3.4, 2 mg/mL of density, spin-coated at 3000 rpm.

To further characterize silver nanowires, a scanning electronic microscopy characterization was performed to describe the distribution of the silver nanowires on the surface of the hybrid MLG/ITO electrode that we get for each doping condition (see Table 1). Figure 3 shows the SEM images of the samples graphene/ITO/Si S3.1, S3.2, S3.3, and S3.4, measured for different concentrations and spin coating speeds of the Ag-NWs: (a) sample S3.1, 1mg/mL of density, spin-coated at 1000 rpm. (b) Sample S3.2, 1mg/mL of density, spin-coated at 3000 rpm. (c) Sample S3.3, 2mg/mL of density, spin-coated at 1000 rpm. (d) Sample S3.4, 2mg/mL of density, spin-coated at 3000 rpm. The density of the deposited nanowires increases by increasing the silver nanowire’s concentration and decreasing the spin coating speed. Figure 3a,c, corresponding to the samples S3.1 and S3.3, which were doped with the concentrations of 1 mg/mL and 2 mg/mL, respectively, at a 1000 rpm spin coating speed, show a good distribution of Ag-NWs. Figure 3b,d, corresponding to the samples S3.2 and S3.4, which were doped with 1 mg/mL and 2 mg/mL of concentration, respectively, spin-coated with a speed of 3000 rpm, show a lower density of deposited Ag-NWs compared to the samples S3.1 and S3.3, doped with the same concentrations of 1 mg/mL and 2 mg/mL, but at a slower spin coating speed (Figure 3a,c). In addition, the samples in Figure 3a,c show more homogeneity in the distribution of the Ag-NWs on the surface. This explains the good optical transmittance with the best sheet resistance achieved with these samples. Finally, the best connection between silver nanowires, achieving a fully communicated network of dopants, is found in the case of 2 mg/mL of concentration and 1000 rpm of spin coating speed (Figure 3c), which explains why the maximum decrease in the sheet resistance was achieved after doping the hybrid graphene/ITO electrode under these conditions, as it will be seen later in the corresponding section. 

**Figure 4:**
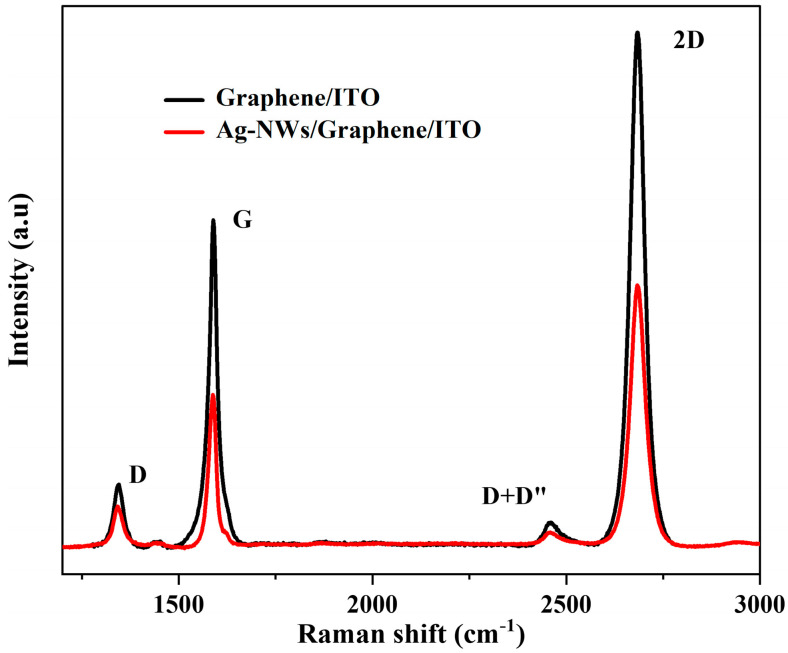
Raman spectra of the CVD graphene after transfer on ITO/Si substrate and annealing at 150 °C for 120 min (black curve) and after doping with Ag-NWs with 2 mg/mL, deposited at 1000 rpm (red curve).

Raman spectroscopy is a fast, nondestructive, high-resolution technique to study the fundamental physical properties of various carbon nanomaterials, including monolayer graphene [62,63,64]. The Raman signature of graphene is characterized by three main bands: D, G, and 2D, through their intensity and line width. The D band is associated with a vibrational breathing mode, only active when material defects or boundaries are present. The G band is characteristic of the in-plane vibration of the carbon in the graphene (sp^2^), and the 2D band is related to the appearance of the Dirac cone in the energy-momentum dispersion, giving insight into the number of layers and the material quality [53,65]. Figure 4) displays the Raman spectra of the CVD graphene used in this work, after transferring to the ITO/Si substrate and annealing at 150 °C for 120 min (black curve) and after doping with Ag-NWs deposited at 1000 rpm and with a density of 2 mg/mL (red curve). The spectra exhibit the characteristic bands of graphene: D, G, and 2D bands, with their central frequencies located at 1342, 1588, and 2687 cm^−1^, respectively. In addition, a peak appears at 2458 cm^−1^ corresponding to the D+D’’ band [66]. Regarding the spectrum after annealing (black curve), the intensity ratio of 2D to G (I_2D_/I_G_) is 2.25, which is quite high, and the 2D peak appears sharp with an FWHM of about 46.5 cm^−1^, and symmetric, indicating that the used graphene consists of one to two layers of graphene [66,67]. The low intensity of the D band gives a ratio of the intensity of D versus the intensity of G (I_D_/I_G_) of 0.21, which means that the graphene has a low defect concentration. After doping the CVD graphene with Ag-NWs (red curve in Figure 4), the new central positions of the D, G, and 2D bands are located at 1340, 1589, and 2682 cm⁻^1^, respectively. Additionally, the appearance of the D+D’ combination band is at 2461 cm⁻^1^. Compared to the spectrum before doping (black curve in Figure 4), the peaks exhibit a slight red shift, which can be attributed to electronic and structural changes and n-doping effects induced by the incorporation of silver nanowires. These alterations highlight the pronounced influence of Ag-NW doping on the graphene’s electronic characteristics, as evident from changes in both the intensity and position of key peaks in the spectral analysis [49]. The ratio of I_2D_/I_G_ for the red curve is approximately 2.4, and the ratio of I_D_/I_G_ is 0.29, which are comparable to the values obtained from the before doping measurement (black curve). This result indicates again the effect of the Ag-NWs on the graphene monolayer.

### 3.3. Doping Density and Spin Coating Parameter Effect on the Electrical and Optical Performance of Hybrid Graphene/ITO Electrode

**Figure 5:**
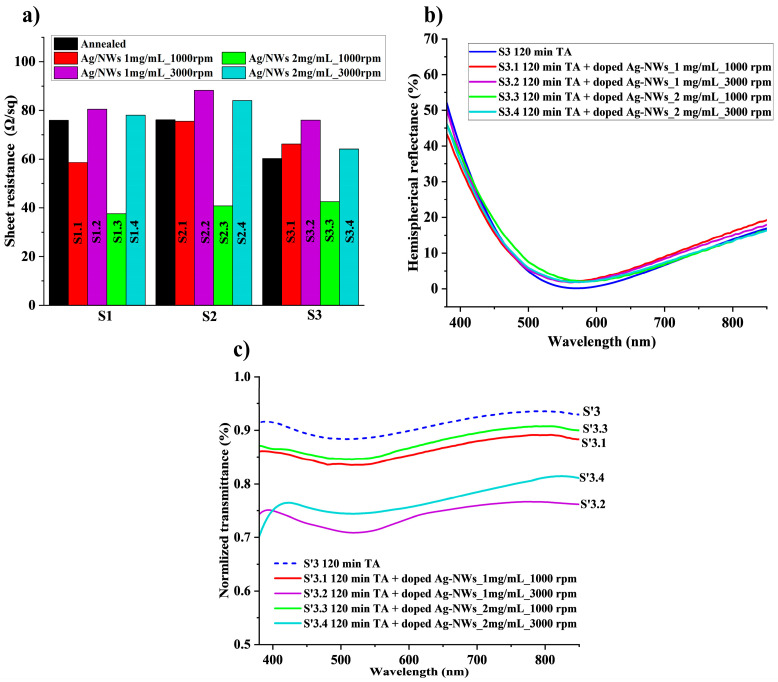
Variation of the electrical and optical characteristics of the Ag-NWs/MLG/ITO electrode with the doping concentration and spin coating speed variation: (**a**) Sheet resistance variation in the sample S3 on the silicon substrate. (**b**) Optical reflectance of the samples S3 on the silicon substrate. (**c**) Normalized optical transmittance of the samples S’3 on the glass substrate.

Ag-NWs are known as a good transparent conductive electrode and a good dopant for graphene, which reduces the sheet resistance of graphene [56,68]. To further enhance the electrical conductivity of the hybrid graphene/ITO electrode, the doping of graphene with Ag-NWs was performed on the samples S1, S2, and S3, deposited on the silicon substrate; and S’1, S’2, and S’3, deposited on the glass substrate (see samples nomenclature provided in Table 1). In this section, a study of the effect of Ag-NWs density on the graphene/ITO hybrid electrodes by controlling the nanowires’ concentration and the spin coating speed is performed. Figure 5a) shows the variation in the sheet resistance at the different doping concentrations and spin coating speeds for the samples on the silicon substrate: S1, S2, and S3, which were annealed at different times and studied in the previous section. The one-minute duration of the spin coating was kept the same for all the samples. Doping the hybrid graphene/ITO electrode with Ag-NWs at a concentration of 2 mg/mL and under spin coating at 1000 rpm improved its sheet resistance from 73.3, 78.0, and 59.6 Ω/sq after the TA treatment for the respective samples S1.3, S2.3, and S3.3 to 37.6, 40.7, and 42.4 Ω/sq. However, samples S1.1, S2.1, and S3.1, doped with 1 mg/mL and spin-coated at 1000 rpm, showed less improvement in the sheet resistance. The sheet resistances of these samples are 58.65, 75.62, and 66.18 Ω/sq (Table S1, Supplementary Information). On the other hand, for the samples, S1.2, S2.2, and S3.2 (density of 1 mg/mL) and S1.4, S2.4, and S3.4 (density of 2 mg/mL) doped with a spin coating at a higher speed of 3000 rpm, the sheet resistance either increased or showed similar values to after annealing (Table S1, Supplementary Information). The enhanced electrical performance of the TCE hybrid can be largely attributed to the density of silver nanowires (Ag-NWs) [48,69]. From the results of this set of experiments, we conclude that the lowest spin coating speed of 1000 rpm and the highest doping concentration of 2 mg/mL provide the best sheet resistance improvement. To further demonstrate the impact of silver nanowires on the performance of the graphene film, we calculated the sheet resistance of the adhered Ag-NWs/graphene composite using Equation (1). Given that the Ag-NWs, graphene, and ITO are configured in a parallel arrangement, the resulting sheet resistance (Rs) of the adhered structure is determined by the equation R_STCE_. This approach allows for a comprehensive understanding of how these materials interact and contribute to the overall conductivity of the film.

(1) $$\frac{1}{{Rs}_{TCE}}=\frac{1}{{Rs}_{ITO}}-\frac{1}{{Rs}_{Ag_{NWs}/Gr}}$$

The sheet resistance of Ag-NWs/graphene is calculated to be 171.16, 257.75, and 158.27 Ω/sq for the samples S3.1, S3.2, and S3.4, respectively (see Table S1 in the supplementary information). where for the best device in this work, S3.3 Rs is 70.06 Ω/sq, with a 95% improvement of the Rs of graphene. The obtained results serve as evidence of the significant role of silver nanowires in enhancing the sheet resistance of graphene. This enhancement is particularly noteworthy, as was found in earlier studies involving GFETs, where similar improvements in sheet resistance were observed on the graphene film. Such advancements highlight the potential of integrating silver nanowires into graphene-based technologies.

Figure 5b depicts the optical reflectance of the hybrid graphene/ITO electrode deposited on the silicon substrate and annealed at 150 °C for 120 min (sample S3). The optical reflectance curve of the doped samples is similar to the curve of the annealed samples (black curve), with a mean optical reflectance of 9.7, 9.6, 9.7, and 9.1% in the range of UV-visible for samples S3.1, S3.2, S3.3, and S3.4, respectively. The reduction in optical transmittance and the rise in the optical reflectance result from the reflective properties of silver nanowires [18,51,70]. Figure 5c represents the optical transmittance of the graphene/ITO hybrid electrode deposited on the glass substrate, sample S’3, annealed at 150 °C for 120 min and doped at different Ag-NWs concentrations and spin coating speed conditions. The optical transmittance decreased after doping with Ag-NWs under all applied conditions. Particularly, the samples where the dopants were deposited at the spin coating speed of 3000 rpm show a higher decrease in the transmittance, compared to the ones where the nanowires were deposited at 1000 rpm. The optical transmittance is about 86.1 and 87.4% for the samples doped with 1 mg/mL (S’3.1) and 2 mg/mL (S’3.3) coated at 1000 rpm, respectively, and about 73.9 and 76.7% for the samples doped with 1 mg/mL (S’3.2) and 2 mg/mL (S’3.4) coated at 3000 rpm, respectively. The normalized optical transmittance of the rest of the samples is presented in Table S2 (Supplementary Information file).

Table 2 summarizes a comparison between the best sheet resistance and optical transmittance obtained in the present work with other similar hybrid electrode configurations studied in other works in the field. Our study demonstrates the delicate balance achieved between low sheet resistance and high optical transmittance facilitated through thermal annealing and the application of a conductive Ag-NWs layer. Comparing the data from the table, our hybrid TCE demonstrates very low sheet resistance, and except for the electrode reported by Liu et al. [25] and the one corresponding to Hemasiri et al. [18], our TCE hybrid electrode demonstrates both better optical and electrical performance.

## 4. Conclusions

In this work, a successful transparent conductive electrode with excellent optical and electrical performance was developed by combining the hybridization of ITO and CVD graphene and the doping of graphene with silver nanowires, known for their high electrical conductivity. The optimization of the fabrication process by applying thermal annealing as a critical step has been verified. By extending the annealing time to 120 min, the electrical conductivity of the hybrid electrode has improved significantly. A reduction in around 45% in sheet resistance is observed, from 107.9 to 59.6 Ω/sq, achieving an optical transmittance of 90.7%. Doping the graphene/ITO hybrid structure with Ag-NWs has further improved its electrical performance, since their incorporation into the structure facilitates better charge transport, reducing the sheet resistance from 59.6 to 42.4 Ω/sq with a reduction in 60%, and maintaining a high optical transmittance of 87.3%. The density of silver nanowires deposited on graphene is controlled by its concentration and the spin coating speed, which are crucial parameters that influence the final properties of the electrode. A lower spin coating speed of 1000 rpm with a high concentration of 2 mg/mL allows for higher coverage and a more uniform distribution of nanowires, leading to better electrical conductivity. A total of 60% is the total reduction in the sheet resistance of the hybrid graphene/ITO doped with Ag-NWs achieved by applying these conditions. These electrodes hold great promise for various applications in optoelectronics, particularly for heterojunction solar cells.

## Figures and Tables

**Table 1:** Summary of the sample nomenclature as a function of the TA and doping conditions used in this work.

	Gr/ITO/Si (S)			Gr/ITO/Glass (S’)		
TA Time Conditions						
15 min TA @ 150 °C	S1			S’1		
60 min TA @ 150 °C	S2			S’2		
120 min TA @ 150 °C	S3			S’3		
Ag-NWs doping conditions (density, spin coating speed, and time)						
1 mg/mL, 1000 rpm, 60 s	S1.1	S2.1	S3.1	S’1.1	S’2.1	S’3.1
1 mg/mL, 3000 rpm, 60 s	S1.2	S2.2	S3.2	S’1.2	S’2.2	S’3.2
2 mg/mL, 1000 rpm, 60 s	S1.3	S2.3	S3.3	S’1.3	S’2.3	S’3.3
2 mg/mL, 3000 rpm, 60 s	S1.4	S2.4	S3.4	S’1.4	S’2.4	S’3.4

**Table 2:** Comparison of the optical and electrical characteristics of the Ag-NW/graphene/ITO hybrid TCE from our work with similar structures obtained from other works.

Structure	R_s_(Ω/sq)	Transmittance (%)	Ref
Ag-NWs/graphene/ITO	42.4	87.3	This work
Graphene/ITO	117	88.66	[18]
ITO nanoparticle-decorated graphene	522.21	85	[51]
Graphene/ITO	78.34	88.25	[25]
Graphene/Ag-NWs/graphene	430	83.2	[1]
ITO(S)/ITO(A)/Graphene	44,2	____	[71]
Ag-NWs/graphene/PET	212.59	82.6	[72]

## Data Availability

The original contributions presented in this study are included in the article. Further inquiries can be directed to the corresponding author.

## Supplementary Materials

The following supporting information can be downloaded at: https://www.mdpi.com/article/10.3390/mi16020204/s1, Figure S1: Histogram of the electrical measurement performed on the graphene used in this work to calculate its sheet resistance. Table S1: Variation of the sheet resistance of TCEs deposited on Si substrate after the deposition of monolayer CVD-graphene, after different TA treatment times, and after different doping conditions with Ag-NWs. Table S2: The average optical transmittance in the visible region (400 to 800 nm) of the samples deposited on glass, after the transfer of graphene, and after the RTA treatment.
