# Supplementary material for: High-Performance Ag-NWs Doped Graphene/ITO Hybrid Transparent Conductive Electrode
Source: Micromachines (Basel). 2025 Feb 11;16(2):204. doi: 10.3390/mi16020204 (PMC11857558; doi:10.3390/mi16020204)
Supplement: Supplementary file 1 [file micromachines-16-00204-s001.zip › micromachines-3430685-supplementary.docx]

# **Supplementary Information**

**High-performance Ag-NWs doped graphene/ITO hybrid transparent conductive electrodes for solar cell applications**

Hana Bourahla ^1,2^, Susana Fernández^3^, Yu Kyoung Ryu^1,4^, Andrés Velasco^1,2^, Chahinez Malkia^1,2^, Alberto Boscá^1,2^, María Belén Gómez-Mancebo^5^, Fernando Calle^1,2^, Javier Martinez^1,6*^

^1^Instituto de Sistemas Optoelectrónicos y Microtecnología (ISOM-UPM), E.T.S.I. de Telecomunicación, Universidad Politécnica de Madrid, Av. Complutense 30, 28040 Madrid, Spain

^2^Departamento de Ingeniería Electrónica, E.T.S.I. de Telecomunicación, Universidad Politécnica de Madrid, Av. Complutense 30, 28040 Madrid, Spain

^3^Departamento de Energía, Centro de Investigaciones Energéticas, Medioambientales y Tecnológicas (CIEMAT), Avda. Complutense 40, Madrid 28040, Spain

^4^Departamento de Física Aplicada e Ingeniería de Materiales, E.T.S.I. Industriales, Universidad Politécnica de Madrid, C/ José Gutiérrez Abascal 2, 28006 Madrid, Spain

^5^División de Química, Centro de Investigaciones Energéticas, Medioambientales y Tecnológicas (CIEMAT), Avda. Complutense 40, Madrid 28040, Spain

^6^Departamento de Ciencia de Materiales, E.T.S.I Caminos, Canales y Puertos, Universidad Politécnica de Madrid. C/ Profesor Aranguren s/n Madrid 28040, Spain

* Corresponding author**:** javier.martinez@upm.es

**S1:** **Graphene sheet resistance**


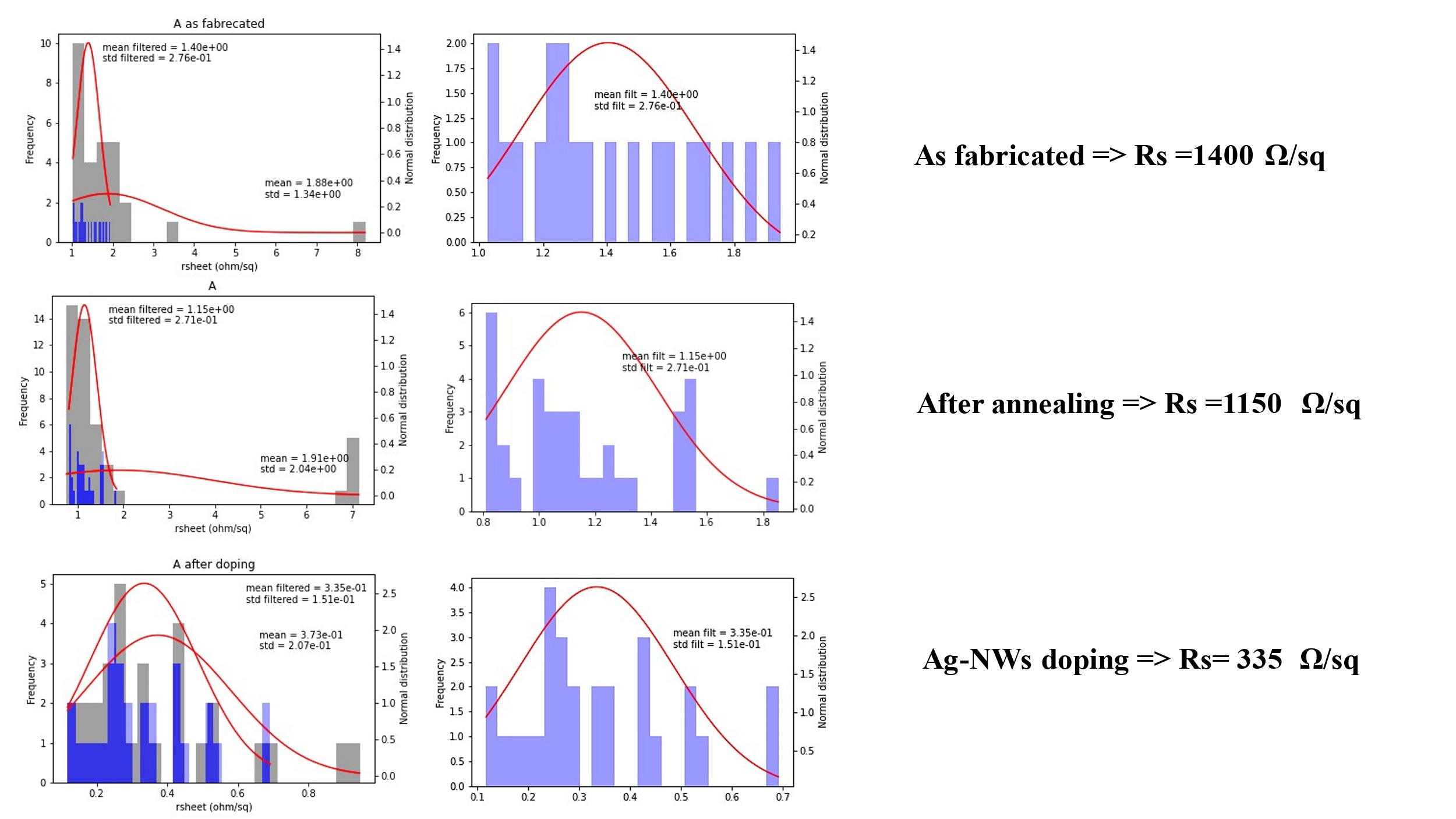


**Figure S1:** Histogram of the electrical measurement performed on the graphene used in this work to calculate its sheet resistance.

**S2: Sheet resistance of graphene/ITO hybrid electrode on Si substrate at different TA times and Ag-NWs doping conditions**

**Table S1:** Variation of the sheet resistance of TCEs deposited on Si substrate after the deposition of monolayer CVD-graphene, after different TA treatment times, and after different doping conditions with Ag-NWs.

| Sheet resistance (Ω/sq) | | | | | |
| --- | --- | --- | --- | --- | --- |
| Sample | ITO/Silicon | Transfer of MLG  (Gr/ITO/Si) | After TA | After Ag-NWs doping  (Ag-NWs/Gr/ITO) | Ag-NWs/Gr  (calculated) |
| S1 TA _15 min | 91.7 | 85.6 | 73.3 (-14% ↓) | S1.1: 58.65  S1.2: 80.52  S1.3: 37.60  S1.4: 78.08 | 162.73  660.44  63.73  525.69 |
| S2 TA _60 min | 100.8 | 90.5 | 78 (-13.8% ↓) | S2.1: 75.62  S2.2: 88.24  S2.3: 40.77  S2.4: 84.07 | 302.72  708.17  68.46  506.53 |
| S3 RTA _120 min | 107.9 | 73.76 | 59.6 (-19% ↓) | S3.1: 66.18  S3.2: 76.06  S3.3: 42.48  S3.4: 64.16 | 171.16  257.75  70.06  158.27 |

**S3: Optical transmittance of the samples graphene/ITO/glass**

**Table S2:** The average optical transmittance in the visible region (400 to 800 nm) of the samples deposited on glass, after the transfer of graphene, and after the RTA treatment.

| Normalized optical transmittance of graphene/ITO/glass doped with Ag-NWs  (%) | | | | |
| --- | --- | --- | --- | --- |
|  | **ITO/glass** | **Gr/ITO/glass** | **Annealed** | **After Ag-NWs doping** |
| **S’1** | 83.29 | 82.23 | 83.43 | S’1.1: 89.4  S’1.2:89.7  S’1.3:91.1  S’1.4:91.6 |
| **S’2** | 83.49 | 82.00 | 87.07 | S’2.1: 89.2  S’2.2: 88.8  S’2.3: 77.1  S’2.4: 89.2 |
| **S’3** | 83.85 | 82.03 | 90.74 | S’3.1: 86.1  S’3.2: 73.9  S’3.3: 87.4  S’3.4: 76.7 |
